# Tempol Ameliorates Psoriatic Skin Inflammation: Evidence of TLR4/NF‐κB/Nrf2 Axis Modulation

**DOI:** 10.1155/mi/5063546

**Published:** 2026-09-12

**Authors:** Sarah Adriana Scuderi, Marika Lanza, Anna Paola Capra, Antonio Catalfamo, Emanuela Esposito, Alessio Ardizzone

**Affiliations:** ^1^ Genetics and Pharmacogenetics Unit, “Gaetano Martino” University Hospital, Messina, Italy; ^2^ Department of Chemical, Biological, Pharmaceutical and Environmental Sciences, University of Messina, Viale Ferdinando Stagno d’Alcontres 31, Messina 98166, Italy, unime.it; ^3^ UniCamillus-Saint Camillus International University of Health Sciences, Via di Sant’Alessandro 8, Rome, Italy

**Keywords:** dermatology, in vitro model, oxidative stress, psoriasis, reconstructed human epidermis (RHE), Tempol

## Abstract

Psoriasis is a chronic inflammatory skin disorder characterized by keratinocyte hyperproliferation, immune dysregulation, oxidative stress, and impaired epidermal barrier function. In the present study, we investigated the protective effects of Tempol, a membrane‐permeable nitroxide with antioxidant and anti‐inflammatory properties, using several psoriasiform models. In HaCaT keratinocytes, stimulation with a cytokine mixture significantly induced inflammatory responses. Tempol (0.5 and 1 mM) showed no cytotoxic effects and markedly reduced the mRNA and protein levels of proinflammatory cytokines, including TNF‐α, IL‐1β, and IL‐6. Tempol was associated with reduced expression of TLR4, MyD88, TRAF6, and nuclear factor‐kappa B (NF‐κB), together with restoration of IκBα expression, findings consistent with modulation of the TLR4/NF‐κB signaling pathway. In parallel, Tempol was associated with activation of the nuclear factor erythroid 2‐related factor 2 (Nrf2)/HO‐1 antioxidant axis, as evidenced by increased expression of Nrf2, HO‐1, and MnSOD, along with a reduction in the pro‐oxidant enzymes NOX2 and NOX4. These effects were associated with decreased oxidative damage, including reduced lipid peroxidation and nitric oxide production. Furthermore, Tempol exerted cytoprotective effects by restoring the balance between pro‐ and antiapoptotic markers, as indicated by increased Bcl‐2 levels, decreased Bax and p53 expression, and a reduced Bax/Bcl‐2 ratio. The anti‐inflammatory activity of Tempol was further confirmed in an imiquimod (IMQ)‐stimulated HaCaT/THP‐1 coculture model, where it significantly reduced the release of key immune mediators, including TLR4, IL‐8, IL‐17A, and IL‐23, highlighting its ability to modulate immune‐epithelial crosstalk. In a reconstructed human epidermis (RHE) model, Tempol preserved tissue viability under psoriasiform conditions and significantly reduced *Staphylococcus aureus* (*S. aureus*) adhesion. Importantly, Tempol restored epidermal barrier integrity, as demonstrated by increased levels of tight junction and differentiation markers, including ZO‐1, Occludin, Claudin‐1, and Filaggrin, even under infection‐associated conditions. Taken as a whole, these findings support the potential of Tempol as a promising therapeutic strategy for psoriasis and related inflammatory skin disorders.

## 1. Introduction

Psoriasis is a chronic, immune‐mediated inflammatory skin disorder affecting ~2%–3% of the global population and characterized by epidermal hyperproliferation, aberrant keratinocyte differentiation, immune dysregulation, and impaired skin barrier function [1–4]. Although traditionally considered a T‐cell‐driven disease, growing evidence indicates that keratinocytes are not merely passive targets of inflammation but actively contribute to disease initiation and progression through the release of cytokines, chemokines, and oxidative mediators [5]. This reciprocal interaction between epidermal cells and immune components amplifies chronic inflammation and sustains the psoriatic phenotype [6, 7]. Current psoriasis treatments include topical therapies, immunosuppressants, and biologics targeting key inflammatory pathways [8]. However, immune‐mediated adverse events, such as psoriasis induced by immune checkpoint inhibitors, and the limitations of current therapies highlight the need for novel treatment strategies with improved efficacy and safety profiles [9, 10]. In this context, Yang et al. [11] evaluated an engineered exosome‐based transdermal therapy as a promising new approach for psoriasis treatment.

At the molecular level, psoriasis is characterized by the activation of multiple inflammatory pathways, including Toll‐like receptor (TLR)‐dependent signaling and nuclear factor‐kappa B (NF‐κB) activation [12, 13]. In particular, TLR4 signaling has emerged as a relevant contributor to psoriasis pathogenesis by promoting MyD88‐dependent downstream activation of TRAF6 and NF‐κB, ultimately leading to increased transcription of proinflammatory mediators such as TNF‐α, IL‐1β, and IL‐6 [14–16]. These cytokines, in turn, contribute to keratinocyte activation, immune cell recruitment, and maintenance of the inflammatory loop [17].

In addition to inflammation, oxidative stress has been increasingly recognized as a key pathogenic driver in psoriasis [18]. Excessive production of reactive oxygen and nitrogen species contributes to lipid peroxidation, protein oxidation, DNA damage, and dysregulation of intracellular signaling pathways [19]. Conversely, endogenous antioxidant defenses, including the nuclear factor erythroid 2‐related factor 2 (Nrf2) pathway, serve as a major protective mechanism against redox imbalance [20, 21]. Upon activation, Nrf2 promotes the expression of cytoprotective enzymes, thereby counteracting oxidative injury [20, 22]. Notably, defective Nrf2 signaling has been associated with psoriasis severity, suggesting that restoration of antioxidant pathways may represent a promising therapeutic strategy [22, 23].

Although psoriasis has not classically been considered a microbiome‐driven disease, altered skin microbial composition and increased risk of *Staphylococcus aureus* (*S. aureus*) infection have been associated with disease severity and barrier dysfunction [24]. Through the production of exotoxins, superantigens, and pathogen‐associated molecular patterns capable of activating TLR signaling, *S. aureus* may further amplify keratinocyte activation, oxidative stress, and proinflammatory cytokine release, thereby sustaining the chronic inflammatory milieu [25]. These observations suggest that microbial aggravation may represent an additional pathogenic component and a relevant factor to consider when evaluating novel protective strategies in psoriasis.

Therefore, pharmacological agents able to modulate inflammation and oxidative stress simultaneously in heterogeneous psoriatic conditions may offer broader skin‐protective effects than therapies targeting single pathways.

Tempol (4‐hydroxy‐2,2,6,6‐tetramethylpiperidine‐N‐oxyl) is a stable, low‐molecular‐weight, membrane‐permeable nitroxide radical that has been extensively investigated for its antioxidant, anti‐inflammatory, and cytoprotective properties [26]. Unlike conventional stoichiometric antioxidants, Tempol can participate in redox‐cycling reactions and exert catalytic antioxidant activity. In particular, it acts as a superoxide dismutase mimetic by promoting the dismutation of superoxide anions while also limiting the formation of highly reactive oxidant species and reducing oxidative damage to lipids, proteins, and nucleic acids [26, 27]. Tempol has additionally been reported to inhibit lipid peroxidation, preserve nitric oxide bioavailability, and reduce the accumulation of reactive oxygen and nitrogen species, thereby contributing to the restoration of cellular redox homeostasis under conditions of sustained oxidative stress [26, 27].

Beyond its direct radical‐scavenging activity, Tempol may influence several redox‐sensitive molecular pathways involved in inflammatory responses [26, 27]. Previous experimental studies have shown that Tempol treatment is associated with reduced activation of NF‐κB‐related signaling, decreased expression of proinflammatory cytokines and chemokines, and attenuation of oxidative enzyme systems, including NADPH oxidases [28–30]. At the same time, Tempol has been reported to enhance endogenous antioxidant responses through modulation of the Nrf2 pathway and downstream antioxidant enzymes, including heme oxygenase‐1 and superoxide dismutase‐related defenses [28–30]. These effects suggest that Tempol may act at the interface between oxidative stress and inflammation, two closely interconnected processes that contribute to the initiation, amplification, and persistence of tissue injury.

Protective activities have been reported in cardiovascular disorders, including hypertension, endothelial dysfunction, ischemia‐reperfusion injury, and cardiac oxidative damage, as well as in neurodegenerative, metabolic, renal, and inflammatory diseases [28–30]. In these settings, Tempol was associated with reduced oxidative stress, suppression of inflammatory mediator production, preservation of mitochondrial and cellular function, and attenuation of tissue damage [28–30]. Its relatively small molecular size and ability to cross biological membranes have also supported interest in its use as a locally administered antioxidant, particularly in tissues in which oxidative and inflammatory damage are spatially restricted [28–30].

These properties are potentially relevant to psoriasis, in which excessive reactive oxygen species production, impaired antioxidant defenses, activation of redox‐sensitive inflammatory pathways, altered keratinocyte homeostasis, and epidermal barrier dysfunction interact to sustain chronic inflammation.

Based on our previous evidence showing that Tempol attenuates inflammation, oxidative stress, and barrier dysfunction in experimental atopic dermatitis through NF‐κB/Nrf2 modulation [31], we hypothesized that similar protective effects may extend to psoriasis, a distinct but partially overlapping inflammatory skin disorder characterized by dysregulated immune signaling, oxidative imbalance, and impaired epidermal barrier integrity. Given the recognized involvement of TLR4 in psoriasis‐associated immune‐inflammatory responses, we further aimed to extend our previous findings by exploring likely Tempol modulation of TLR4‐driven signaling, a mechanism not previously investigated in our dermatological studies. To match this aim, we evaluated the protective effects of Tempol in complementary in vitro psoriasis‐relevant models, including keratinocyte monocultures and immune coculture systems as well as reconstructed human epidermis (RHE) with or without bacterial challenge. In particular, because psoriasis is a multifactorial disease involving distinct cellular and tissue‐level mechanisms, no single in vitro model fully reproduces its complexity. Therefore, complementary experimental systems employing validated cytokine induction protocols appropriate for each model were used to investigate whether the protective effects of Tempol were maintained across different psoriasis‐relevant settings rather than to directly compare responses elicited by different cytokine cocktails.

## 2. Materials and Methods

### 2.1. In Vitro Study

#### 2.1.1. Materials and Cell Culture Conditions

Tempol and all other chemicals and reagents used in this study were obtained from Sigma–Aldrich (Milan, Italy), unless otherwise stated. The experiments were conducted using the highest purity grade available for each compound.

Human HaCaT keratinocytes were obtained from CLS Cell Lines Service GmbH, Germany [32]. The official cell line name is HaCaT, and the Research Resource Identifier is RRID:CVCL_0038. The cell line is of human origin (*Homo sapiens*), male sex, and skin/epidermal keratinocyte origin. Cells were cultured in Dulbecco’s modified Eagle’s medium (DMEM) supplemented with 10% fetal bovine serum (FBS) and 1% penicillin‐streptomycin in a humidified incubator at 37°C with 5% CO_2_. The cell line was not reported as misidentified or contaminated according to the available cell line databases. Cells were confirmed to be free of mycoplasma contamination before the experiments. A psoriasis‐like keratinocyte model was established by adding an M5 cytokine cocktail composed of IL‐17A, IL‐22, oncostatin M, IL‐1α, and TNF‐α (Thermo Fisher Scientific), each at a final concentration of 2.5 ng/mL, to the culture medium of HaCaT keratinocytes, as previously described [33, 34].

#### 2.1.2. Cell Viability Assay

Cell viability was measured using a mitochondria‐dependent dye for live cells (tetrazolium dye; MTT), as previously described [35, 36]. For the first screening, HaCaT cells were seeded in 96‐well plates at a density of ~10,000 cells per well and allowed to adhere for 24 h. Cells were then treated with increasing concentrations of Tempol (0.1, 0.2, 0.5, and 1 mM) for 24 h to identify noncytotoxic concentrations. The concentrations of Tempol were chosen according to a previous in vitro study [37].

For the inflammatory model, HaCaT keratinocytes were seeded in 12‐well plates and treated with Tempol (0.1, 0.2, 0.5, and 1 mM) for 2 h and subsequently stimulated with M5 cytokines (2.5 ng/mL) for 48 h.

Thereafter, in both cases, cells were incubated at 37°C with MTT solution (0.2 mg/mL) for 1 h. The resulting formazan crystals were dissolved using dimethyl sulfoxide (DMSO), and absorbance was measured at 570 nm using a microplate reader. Cell viability was expressed as a percentage relative to untreated control cells.

#### 2.1.3. ELISA Assays

Using the same methodology and timing described in 2.2.2 to treat cells, the levels of TNF‐α (#MBS455676), IL‐1β (#MBS2021180), and IL‐6 (#MBS2707978) were measured in HaCaT cell supernatants according to the manufacturer’s instructions [36].

Protein levels of IκBα (#MBS450066), NF‐κB (#MBS2703751), TLR4 (#MBS2024506), MyD88 (#MBS701532), TRAF6 (#MBS451749), Nrf2 (#MBS744368), MnSOD (#MBS453584), HO‐1 (#MBS2024044), NOX2 (#MBS1608003), and NOX4 (#MBS901457) were evaluated in HaCaT cell lysates using specific human ELISA kits, according to the manufacturers’ instructions.

Also, Bax (#MBS162710), Bcl‐2 (#MBS450833), and p53 (#MBS355295) levels were assessed by ELISA assays in cell lysates.

Briefly, cell lysates and supernatants were collected after treatments, added to ELISA plates precoated with specific antibodies, and incubated following the protocols provided by the manufacturer. After the addition of substrate solution, absorbance was measured at 450 nm using a microplate reader.

Prior to the ELISA, the total protein concentration was determined using the Bradford assay. Protein levels were measured to normalize sample loading and reduce potential bias arising from variations in the total protein content across samples.

#### 2.1.4. Quantitative Reverse Transcription Polymerase Chain Reaction (qRT‐PCR)

For qRT‐PCR analyses, total RNA from HaCaT cells was isolated using the RNeasy Mini Isolation Kit (Qiagen, Germantown, MD, USA) and quantified following the standard protocol [37]. qRT‐PCR analyses were performed on three independent experiments; each sample was tested in triplicate, and gene expression was measured using the 2^−ΔΔCt^ method. RNA from control cells was used as a reference for relative expression quantification. Specific primers were used and described in Table S1: “List of primers used for RT‐qPCR.” The reference gene selected was glyceraldehyde 3‐phosphate dehydrogenase (GAPDH).

#### 2.1.5. Malondialdehyde (MDA) Assay

HaCaT cells (1 × 10^5^ cells/well) were seeded in six‐well plates and treated as in a previous study [37]. At the end of the experimental treatments, cells were collected to determine MDA levels as an index of lipid peroxidation using a commercially available MDA assay kit according to the manufacturer’s instructions. The assay is based on the reaction between MDA and thiobarbituric acid (TBA), generating a colored adduct that was quantified spectrophotometrically at 532 nm.

#### 2.1.6. Measurement of Nitrite (${\text{NO}}_{2}^{-}$) Levels

Nitrite production, as an indirect indicator of nitric oxide generation, was assessed using the colorimetric Griess reaction [38]. HaCaT cells were seeded and treated as described above. Following experimental treatments, cell culture supernatants were collected, and nitrite (${\text{NO}}_{2}^{-}$) levels released into the medium were determined. Briefly, 100 μL of each supernatant was transferred into microplate wells and mixed with an equal volume of Griess reagent, consisting of 1% sulfanilamide and 0.1% N‐naphthylethylenediamine dihydrochloride prepared in 5% phosphoric acid. After incubation for 10 min at room temperature, the absorbance was measured at 540 nm using a microplate reader. Nitrite concentrations were calculated from a standard curve generated using sodium nitrite standards.

#### 2.1.7. Western Blot

Western blot analysis was performed on HaCaT cells to evaluate the protein expression of antinuclear factor of kappa‐light‐chain‐enhancer in B cells (NF‐κB) (1:500; Santa Cruz Biotechnology, Dallas, TX, USA; sc‐8008), inhibitor nuclear factor of kappa‐light‐chain‐enhancer in B cells α (IκBα) (1:500; Santa Cruz Biotechnology, Dallas, TX, USA; sc‐1643), and Nrf2 (1:500; Santa Cruz Biotechnology, Dallas, TX, USA; sc‐365949), as previously described [39]. GAPDH (1:1000; Santa Cruz Biotechnology; Dallas, TX, USA, sc‐365062 HRP) was used as the loading control. ImageJ software was used to quantify the protein expression.

### 2.2. Materials and Coculture Study

Unless otherwise specified, Tempol, imiquimod (IMQ), and all other chemicals and reagents employed in this study were purchased from Sigma–Aldrich (Milan, Italy). All experimental procedures were performed using reagents of the highest commercially available purity.

For HaCaT–THP‐1 cocultures, ELISA assays were performed as previously described [40]. Human HaCaT keratinocytes and human THP‐1 monocytic cells were used to establish the coculture model. HaCaT cells were obtained from CLS Cell Lines Service GmbH, Germany. The official cell line name is HaCaT, and the Research Resource Identifier is RRID:CVCL_0038. HaCaT cells are of human origin (*Homo sapiens*), male sex, and skin/epidermal keratinocyte origin. The human leukemia monocytic cell line THP‐1 was purchased from the American Type Culture Collection (ATCC; [Rockville, MD, USA/Manassas, VA, USA]). The official cell line name is THP‐1, and the Research Resource Identifier is RRID:CVCL_0006. THP‐1 cells are of human origin (*Homo sapiens*) and male sex and were derived from the peripheral blood of a patient with acute monocytic leukemia. THP‐1 cells were cultured in RPMI 1640 (Sigma–Aldrich, St. Louis, MO, USA) containing 10% FBS and 1% penicillin/streptomycin at 37°C and 5% CO_2_.

Both cell lines were checked against available cell line databases and were not reported as misidentified or contaminated. HaCaT and THP‐1 cells were confirmed to be free of mycoplasma contamination before the described experiments.

A Transwell coculture system consisting of 12‐well inserts fitted with 0.4 μm pore‐size polycarbonate membranes was used. HaCaT cells were seeded in the apical compartment at a density of 3 × 10^5^ cells per insert, while 1 mL of culture medium was added to the basolateral compartment. After 6 h, THP‐1 cells were introduced at a density of 3 × 10^4^ cells per insert, corresponding to 10% of the HaCaT cell number, to establish the coculture model [40]. Upon formation of a confluent cell layer, the medium was replaced, and cultures were exposed to 1 μM IMQ, either alone or in combination with Tempol at 0.5 or 1 mM. The concentration of IMQ (1 μM) was chosen based on previous studies [40, 41]. Untreated cocultures served as controls. Following treatment, TLR4 levels were measured in cell lysates using human ELISA kits, whereas IL‐8 (#MBS2019724), IL‐17A (#MBS2887591), and IL‐23 (#MBS2023650) concentrations were quantified in culture supernatants according to the manufacturers’ protocols.

### 2.3. RHE Study Protocol

#### 2.3.1. RHE SkinEthic RHE

Except where otherwise indicated, all chemicals and reagents, including Tempol, were obtained from Sigma–Aldrich (Milan, Italy). Only reagents of the highest purity grade commercially available were used for the experimental procedures.

The SkinEthic RHE model, supplied by EPISKIN Laboratories (Lyon, France, EU), is a well‐established in vitro system composed of normal human keratinocytes. This 3D epidermal model reproduces the key structural layers of human skin, including the basal layer, stratum spinosum, stratum granulosum, and stratum corneum, and is cultured at the air–liquid interface on a 0.5 cm^2^ polycarbonate filter using a chemically defined medium, as previously reported [42]. The Episkin model is the only validated stand‐alone alternative to animal‐based skin irritation testing, having demonstrated high accuracy, sensitivity, and specificity in toxicological and cosmetic testing [42]. Despite the physiological relevance of skin organotypic cultures derived from human biopsies, their broader use is limited by issues such as donor variability, restricted tissue availability, and ethical constraints [42]. Compared to other 3D skin models, like collagen‐based hydrogels, which often suffer from limited stability and reproducibility, the RHE system offers notable advantages, including structural similarity to the native epidermis, consistent stratification, and long‐term viability under experimental conditions.

#### 2.3.2. *Staphylococcus aureus* Culture

For experimental infection, *Staphylococcus aureus* (*S. aureus*) was cultured in brain‐heart infusion (BHI) broth at 37 °C under constant agitation until reaching the exponential growth phase (~1 × 10^9^ colony forming unit [CFU]/mL), as previously done [42]. Bacterial cells were then collected by centrifugation at 5000 × g for 5 min, washed three times with phosphate‐buffered saline (PBS), and finally resuspended in fresh medium to obtain the desired concentration for infection assays.

#### 2.3.3. Psoriasis Model Induction

Psoriasis induction was established as previously reported [42, 43]. SkinEthic RHE tissues were stimulated with a proinflammatory cytokine cocktail containing IL‐1α (10 ng/mL), IL‐6 (5 ng/mL), TNF‐α (5 ng/mL), and IL‐17A (10 ng/mL) to stimulate a psoriatic‐like phenotype over a 24 h period. Following this induction phase, the RHE tissues were treated with Tempol, prepared in culture medium at final concentrations of 0.1, 0.2, 0.5, and 1 mM, and incubated for an additional 24 h at 37 °C in a humidified atmosphere containing 5% CO_2_.

#### 2.3.4. *S. aureus* Infection Associated‐Psoriasis Model

RHE cells, following cytokine mix treatment, were infected with *S. aureus* for 90 min to aggravate psoriasis disease (5 × 10^7^ bacteria/well) [42, 44].

After, RHE cells were treated with Tempol at concentrations of 0.1, 0.2, 0.5, and 1 mM and incubated at 37°C with 5% CO_2_ for 24 h.

#### 2.3.5. Experimental Groups

For the study, the following experimental groups were established:

Control group (CTR): untreated RHE cells.
RHE cells were treated with SDS 1% for 24 h.
Tempol 0.1 mM: RHE cells were treated with Tempol 0.1 mM for 24 h.
Tempol 0.2 mM: RHE cells were treated with Tempol 0.2 mM for 24 h.
Tempol 0.5 mM: RHE cells were treated with Tempol 0.5 mM for 24 h.
Tempol 1 mM: RHE cells were treated with Tempol 1 mM for 24 h.
Cytokines group: RHE was treated with cytokine mix for 24 h.
Cytokines + Tempol 0.1 mM group: RHE was treated with cytokine mix and Tempol 0.1 mM for 24 h.
Cytokines + Tempol 0.2 mM group: RHE was treated with cytokine mix and Tempol 0.2 mM for 24 h.
Cytokines + Tempol 0.5 mM group: RHE was treated with cytokine mix and Tempol 0.5 mM for 24 h.
Cytokines + Tempol 1 mM group: RHE was treated with cytokine mix and Tempol 1 mM for 24 h.
Cytokines + *S. aureus* infection group: RHE was treated with cytokine mix and *S. aureus*.
Cytokines + *S. aureus* infection + Tempol 0.1 mM group: RHE was treated with cytokine mix, *S. aureus*, and Tempol 0.1 mM.
Cytokines + *S. aureus* infection + Tempol 0.2 mM group: RHE was treated with cytokine mix, *S. aureus*, and Tempol 0.2 mM.
Cytokines + *S. aureus* infection + Tempol 0.5 mM group: RHE was treated with cytokine mix, *S. aureus*, and Tempol 0.5 mM.
Cytokines + *S. aureus* infection + Tempol 1 mM group: RHE was treated with cytokine mix, *S. aureus*, and Tempol 1 mM.

RHE cells, except the control group, were treated with cytokine mix for 24 h and then with Tempol at different concentrations for the other 24 h. For the *S. aureus* infection, following psoriasis model induction, RHE cells were infected with *S. aureus* for 90 min and then treated with Tempol at different concentrations for 24 h.

#### 2.3.6. Cell Viability in RHE

Cell viability was assessed using the MTT assay based on the enzymatic conversion of MTT (3‐[4,5‐dimethylthiazol‐2‐yl]‐2,5‐diphenyltetrazolium bromide) into insoluble formazan crystals by mitochondrial dehydrogenases. SkinEthic RHE tissues were incubated with 0.3 mL of MTT solution (1 mg/mL) for 3 h at 37°C. Following incubation, 1.5 mL of isopropanol was added to solubilize the formazan crystals over a 2 h extraction period. An aliquot of 200 μL from each extract was transferred in triplicate to a 96‐well plate, and absorbance was measured at 570 nm using a spectrophotometer to quantify the cell metabolic activity [42].

#### 2.3.7. CFU Evaluation

To assess bacterial adhesion, serial dilutions of the RHE culture supernatants were plated onto BHI agar plates and incubated overnight at 37°C. The number of adhered bacteria was determined by counting CFUs, and the adhesion rate was calculated as the ratio between the CFUs of adherent bacteria and the CFUs corresponding to the initial bacterial inoculum [42].

#### 2.3.8. ELISA Kits

ELISA kits were used to quantify the levels of ZO‐1 (#MBS700684), Occludin (#MBS903313), Filaggrin (#MBS944540), and Claudin‐1 (#MBS2021065) in RHE lysates. All assays were performed according to the manufacturers’ instructions, following previously validated procedures [36]. Prior to the ELISA, the total protein concentration was determined using the Bradford assay. Protein levels were measured to normalize sample loading and reduce potential bias arising from variations in the total protein content across samples.

#### 2.3.9. Transepithelial Electrical Resistance (TEER) Measurements

At the end of experimental procedures, TEER was performed as previously described [45]. For TEER measurements, a Millicell ERS 3.0 Digital Voltohmmeter (Merck) was employed. Experiments were performed on triplicate tissues, and results are expressed as mean ± standard deviation (SD).

### 2.4. Statistical Analysis

Data normality was assessed using the Shapiro–Wilk test prior to performing one‐way analysis of variance (ANOVA), followed by Bonferroni’s post hoc test for multiple comparisons. Data are presented as the mean ± SD from three independent experiments. All analyses were conducted using GraphPad Prism 9.0, and differences were considered statistically significant at *p* < 0.05.

## 3. Results

### 3.1. Tempol Attenuated M5‐Induced Inflammatory Responses Without Affecting Cell Viability

First, to assess the potential cytotoxicity of Tempol in the HaCaT cell line and identify noncytotoxic concentrations for subsequent experiments, cell viability was evaluated by the MTT assay.

**Figure 1:**
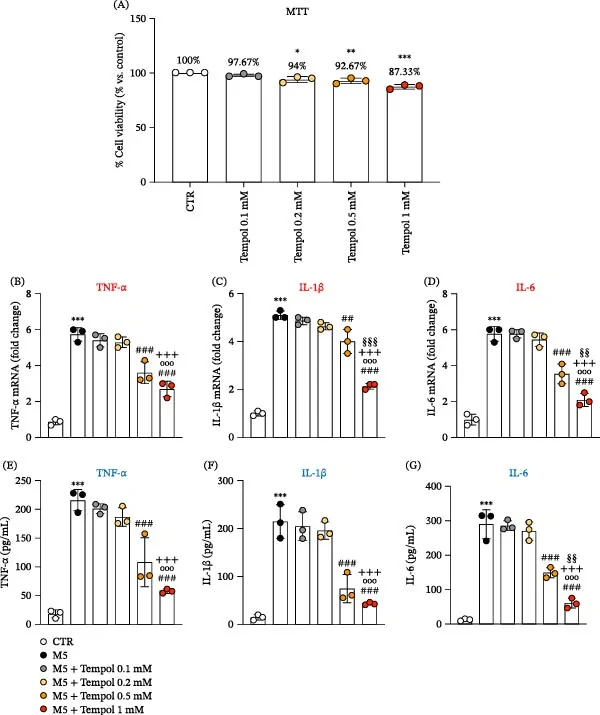
Cell viability was assessed by MTT assay after treatment with increasing concentrations of Tempol (0.1, 0.2, 0.5, and 1 mM) (A). mRNA expression levels of TNF‐α, IL‐1β, and IL‐6 were evaluated by qRT‐PCR in M5‐stimulated HaCaT cells treated with Tempol (B–D). Protein levels of TNF‐α, IL‐1β, and IL‐6 were measured by ELISA (E–G). Data are presented as mean ± SD from three independent experiments. One‐way ANOVA followed by Bonferroni’s post hoc test.  ^∗^ *p* < 0.05 vs. CTR;  ^∗∗^ *p* < 0.01 vs. CTR;  ^∗∗∗^ *p* < 0.001 vs. CTR; ^##^ *p* < 0.01 and ^###^ *p* < 0.001 vs. M5; ^°°°^ *p* < 0.001 vs. M5 + Tempol 0.1 mM; ^+++^ *p* < 0.001 vs. M5 + Tempol 0.2 mM; ^§§^ *p* < 0.01 vs. M5 + Tempol 0.5 mM; ^§§§^ *p* < 0.001 vs. M5 + Tempol 0.5 mM.

Tempol did not exert relevant cytotoxic effects at concentrations ranging from 0.1 to 1 mM, with cell viability remaining at 87.33% even at the highest concentration tested (Figure 1A).

In another experiment, exposure to M5 markedly increased the mRNA expression of proinflammatory cytokines, including TNF‐α, IL‐1β, and IL‐6, compared with that of CTR (Figure 1B–D). Treatment with Tempol at lower concentrations (0.1 and 0.2 mM) did not substantially affect M5‐induced cytokine expression, whereas higher concentrations (0.5 and 1 mM) significantly reduced TNF‐α, IL‐1β, and IL‐6 mRNA levels in a concentration‐dependent manner (Figure 1B–D). In particular, the strongest inhibitory effect was observed at 1 mM Tempol, where cytokine expression was markedly suppressed compared with M5‐treated cells (Figure 1B–D).

Consistent with transcriptional findings, protein levels of TNF‐α, IL‐1β, and IL‐6 were significantly elevated following M5 exposure and were reduced by Tempol treatment (Figure 1E–G). Again, the anti‐inflammatory effect was more evident at 0.5 and 1 mM, with 1 mM producing the greatest reduction in cytokine release (Figure 1E–G). Since the two lowest concentrations tested (0.1 and 0.2 mM) did not produce significant anti‐inflammatory effects, they were excluded from subsequent experiments, and only 0.5 and 1 mM Tempol were selected for further investigation.

### 3.2. Tempol Modulates the Expression of TLR4/NF‐κB Signaling Pathway in M5‐Stimulated HaCaT Cells

To investigate the molecular mechanisms underlying the anti‐inflammatory effects of Tempol, the expression of key mediators involved in the TLR4/NF‐κB signaling pathway was evaluated.

**Figure 2:**
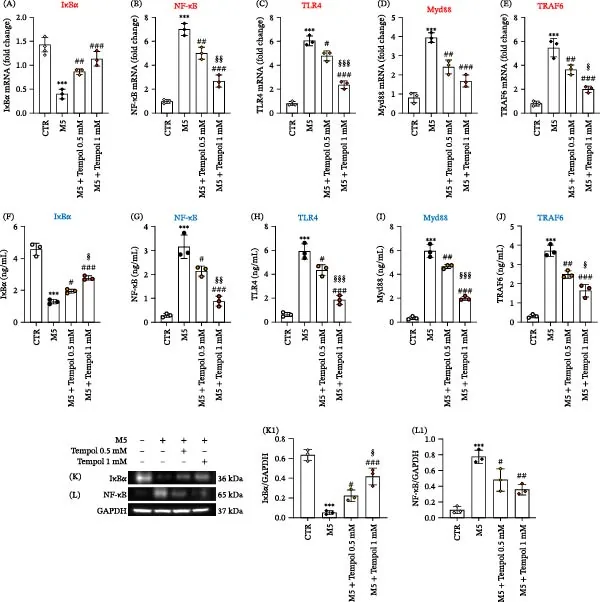
Effects of Tempol on M5‐induced TLR4/NF‐κB signaling in HaCaT cells. mRNA expression levels of IκBα, NF‐κB, TLR4, MyD88, and TRAF6 were evaluated by qRT‐PCR in M5‐stimulated HaCaT cells treated with Tempol (0.5 and 1 mM) (A–E). Protein levels of IκBα, NF‐κB, TLR4, MyD88, and TRAF6 were measured by ELISA (F–J). Western blot analysis for IκBα and NF‐κB was performed in HaCaT cells (K, K1 and L, L1). Data are presented as mean ± SD from three independent experiments. One‐way ANOVA followed by Bonferroni’s post hoc test.  ^∗∗∗^ *p* < 0.001 vs. Control; ^#^ *p* < 0.05, ^##^ *p* < 0.01, ^###^ *p* < 0.001 vs. M5; ^§^ *p* < 0.05 vs. M5 + Tempol 0.5 mM; ^§§^ *p* < 0.01 vs. M5 + Tempol 0.5 mM; ^§§§^ *p* < 0.001 vs. M5 + Tempol 0.5 mM.

M5 stimulation significantly increased the mRNA expression of NF‐κB, TLR4, MyD88, and TRAF6 while reducing IκBα levels compared with those of CTR cells (Figure 2A–E). Treatment with Tempol significantly counteracted these effects, restoring IκBα expression and reducing NF‐κB, TLR4, MyD88, and TRAF6 levels in a concentration‐dependent manner, with the strongest effect observed at 1 mM (Figure 2A–E).

In line with gene expression data, protein levels of NF‐κB, TLR4, MyD88, and TRAF6 were markedly increased following M5 stimulation, whereas IκBα levels were reduced (Figure 2F–J). Tempol treatment significantly reversed these alterations, increasing IκBα and suppressing the M5‐induced upregulation of NF‐κB, TLR4, MyD88, and TRAF6, with greater efficacy at 1 mM than at 0.5 mM (Figure 2F–J). Data about IκBα and NF‐κB were also confirmed by Western blot analysis as shown in Figure 2K, K1, L, L1.

### 3.3. Tempol Modulates the Expression of Nrf2/HO‐1 and NOX‐Related Oxidative Stress Markers in M5‐Stimulated HaCaT Cells

**Figure 3:**
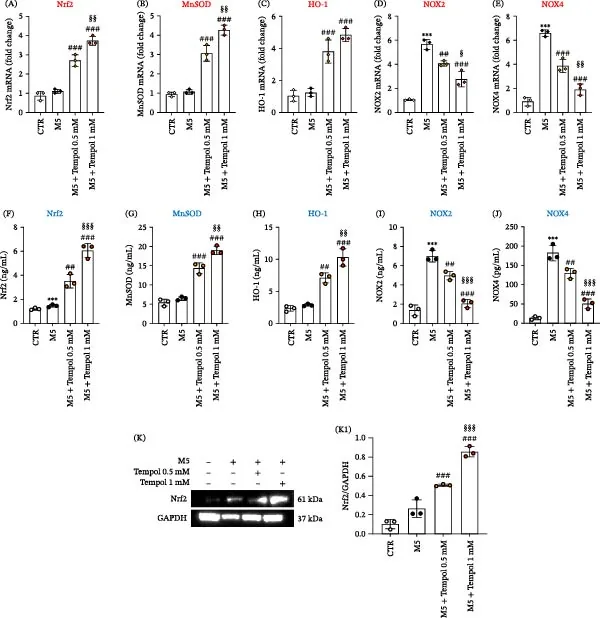
Effects of Tempol on oxidative stress‐related markers in M5‐stimulated HaCaT cells. mRNA expression levels of Nrf2, MnSOD, HO‐1, NOX2, and NOX4 were evaluated by qRT‐PCR in M5‐stimulated HaCaT cells treated with Tempol (0.5 and 1 mM) (A–E). Protein levels of Nrf2, MnSOD, HO‐1, NOX2, and NOX4 were measured by ELISA (F–J). Western blot analysis for Nrf2 was performed in HaCaT cells (K, K1). Data are presented as mean ± SD from three independent experiments. One‐way ANOVA followed by Bonferroni’s post hoc test.  ^∗∗∗^ *p* < 0.001 vs. CTR; ^##^ *p* < 0.01, ^###^ *p* < 0.001 vs. M5; ^§^ *p* < 0.05 vs. M5 + Tempol 0.5 mM; ^§§^ *p* < 0.01 vs. M5 + Tempol 0.5 mM; ^§§§^ *p* < 0.001 vs. M5 + Tempol 0.5 mM.

Given the known antioxidant properties of Tempol, we next investigated whether its protective effects were associated with the modulation of redox homeostasis. Analysis of antioxidant defense markers showed that Tempol enhanced the expression of Nrf2, MnSOD, and HO‐1 at both transcriptional and protein levels, with a stronger effect at 1 mM than at 0.5 mM (Figure 3A–C, F–H, respectively).

In parallel, the expression of the pro‐oxidant enzymes NOX2 and NOX4, elevated under M5 stimulation, was significantly reduced by Tempol treatment (Figure 3D, E, I, J, respectively). Western blot analysis further confirmed the increase in total Nrf2 protein expression observed following Tempol treatment (Figure 3K, K1). However, since subcellular fractionation was not performed, these data do not provide direct evidence of Nrf2 nuclear translocation.

### 3.4. Tempol Attenuates Lipid Peroxidation and Nitric Oxide Production in M5‐Stimulated HaCaT Cells

To further confirm the antioxidant activity of Tempol, markers of oxidative damage were evaluated in M5‐stimulated HaCaT cells.

**Figure 4:**
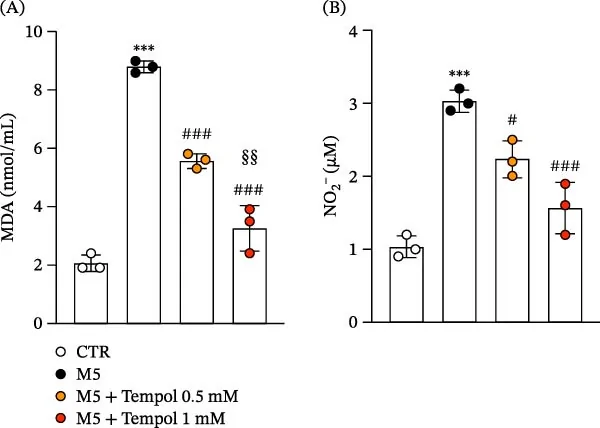
Effects of Tempol on MDA and ${\text{NO}}_{2}^{-}$ levels in M5‐stimulated HaCaT cells. Levels of MDA (A) and nitric oxide (${\text{NO}}_{2}^{-}$) (B) were measured in M5‐stimulated HaCaT cells treated with Tempol (0.5 and 1 mM). Data are presented as mean ± SD from three independent experiments. One‐way ANOVA followed by Bonferroni’s post hoc test.  ^∗∗∗^ *p* < 0.001 vs. CTR; ^#^ *p* < 0.05, ^###^ *p* < 0.001 vs. M5; ^§§^ *p* < 0.01 vs. M5 + Tempol 0.5 mM.

M5 stimulation significantly increased MDA levels compared with CTR, indicating enhanced lipid peroxidation (Figure 4A). Tempol treatment significantly reduced MDA levels at both tested concentrations, with a greater reduction observed at 1 mM than at 0.5 mM (Figure 4A).

Similarly, nitric oxide production (${\text{NO}}_{2}^{-}$), markedly elevated following M5 stimulation, was significantly attenuated by Tempol treatment (Figure 4B). Also, in this case, the inhibitory effect was more pronounced at 1 mM (Figure 4B).

### 3.5. Tempol Modulates Apoptosis‐Related Markers in M5‐Stimulated HaCaT Cells

Apoptosis‐related markers were next evaluated to determine whether Tempol contributed to cell protection beyond its anti‐inflammatory and antioxidant effects.

**Figure 5:**
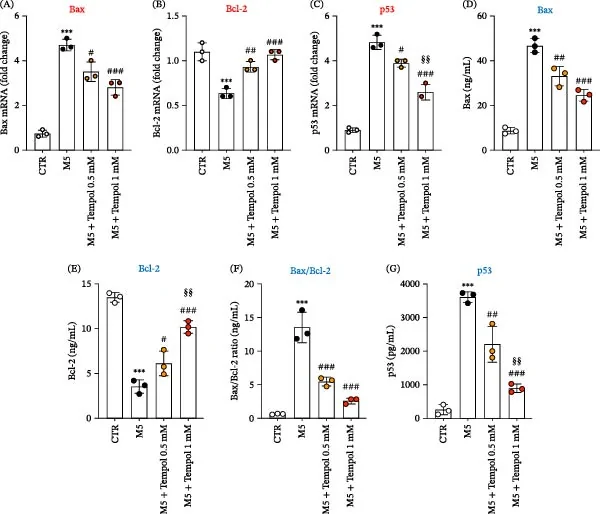
Effects of Tempol on apoptosis‐related markers in M5‐stimulated HaCaT cells. mRNA expression levels of Bcl‐2, Bax, and p53 were evaluated by qRT‐PCR in M5‐stimulated HaCaT cells treated with Tempol (0.5 and 1 mM) (A–C). Protein levels of Bcl‐2, Bax, and p53 were measured by ELISA (D, E, and G). Bax/Bcl‐2 ratio was also determined (F). Data are presented as mean ± SD from three independent experiments. One‐way ANOVA followed by Bonferroni’s post hoc test.  ^∗∗∗^ *p* < 0.001 vs. CTR; ^#^ *p* < 0.05, ^##^ *p* < 0.01, ^###^ *p* < 0.001 vs. M5; ^§§^ *p* < 0.01 vs. M5 + Tempol 0.5 mM.

M5 stimulation significantly reduced the expression of the antiapoptotic marker Bcl‐2 while increasing the proapoptotic markers such as Bax and p53 at both mRNA (Figure 5A–C) and protein levels (Figure 5D, E, G) compared with CTR. Tempol treatment significantly reversed these alterations, restoring Bcl‐2 expression and reducing Bax and p53 levels, with greater efficacy observed at 1 mM than at 0.5 mM (Figure 5A–E, G).

Consistent with these findings, the Bax/Bcl‐2 ratio, markedly increased by M5 stimulation, was significantly reduced following Tempol treatment (Figure 5F), indicating a shift toward a more prosurvival profile.

### 3.6. Tempol Attenuates Immune‐Inflammatory Mediators Release in IMQ‐Stimulated HaCaT/THP‐1 Coculture

**Figure 6:**
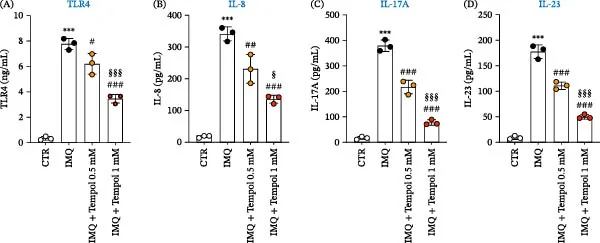
Effects of Tempol on inflammatory mediators in IMQ‐stimulated HaCaT/THP‐1 coculture. Protein levels of TLR4 (A), IL‐8 (B), IL‐17A (C), and IL‐23 (D) were measured by ELISA in IMQ‐stimulated HaCaT/THP‐1 cocultures treated with Tempol (0.5 and 1 mM). Data are presented as mean ± SD from three independent experiments. One‐way ANOVA followed by post hoc test.  ^∗∗∗^ *p* < 0.001 vs. CTR; ^#^ *p* < 0.05, ^##^ *p* < 0.01, ^###^ *p* < 0.001 vs. IMQ; ^§^ *p* < 0.05 vs. IMQ + Tempol 0.5 mM; ^§§§^ *p* < 0.001 vs. IMQ + Tempol 0.5 mM.

To further evaluate the anti‐inflammatory effects of Tempol in a more physiologically relevant model, a HaCaT/THP‐1 coculture system stimulated with IMQ was employed. IMQ significantly increased the levels of TLR4, IL‐8, IL‐17A, and IL‐23 compared with CTR, confirming amplification of inflammatory signaling under coculture conditions (Figure 6A–D).

Also, in this set of experiments, Tempol treatment significantly reduced TLR4 levels, with a more pronounced effect observed at 1 mM than at 0.5 mM (Figure 6A). A comparable inhibitory effect was observed for the psoriasis‐associated cytokines IL‐8, IL‐17A, and IL‐23, both significantly reduced following Tempol exposure at both tested concentrations (Figure 6B–D).

### 3.7. Tempol Preserves Cell Viability and Reduces Bacterial Adhesion in a Psoriasiform RHE Model

Finally, the protective effects of Tempol were investigated using a RHE model to assess its activity in a three‐dimensional psoriasiform setting.

**Figure 7:**
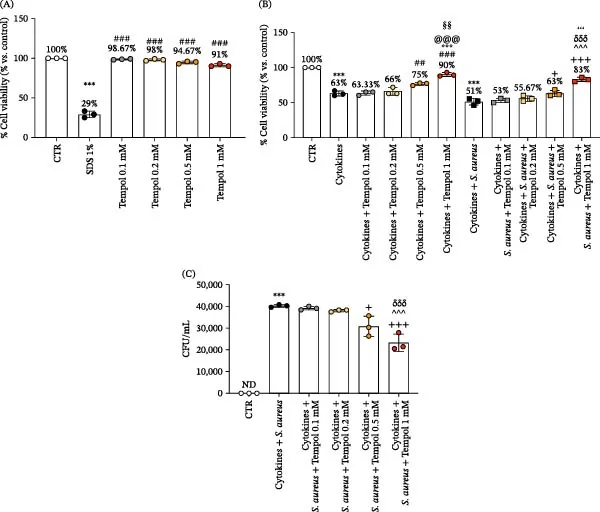
Effects of Tempol on cell viability and bacterial adhesion in psoriasiform RHE. Cell viability was evaluated by MTT assay in SkinEthic RHE treated with Tempol at different concentrations to assess cytotoxicity (A). The effects of Tempol (0.1, 0.2, 0.5, and 1 mM) on cell viability were further assessed in cytokine‐induced psoriasiform RHE and in the *S. aureus*‐associated psoriasis model (B). Bacterial adhesion was quantified as CFU of adhered bacteria relative to the initial bacterial inoculum (C). Data are presented as mean ± SD from three independent experiments. One‐way ANOVA followed by Bonferroni’s post hoc test. In Figure 1A:  ^∗∗∗^ *p* < 0.001 vs. CTR and ^###^ *p* < 0.001 vs. SDS 1%; In Figure 1B, C:  ^∗∗∗^ *p* < 0.001 vs. CTR; ^##^ *p* < 0.01 and ^###^ *p* < 0.001 vs. cytokines; ^+^ *p* < 0.05 and ^+++^ *p* < 0.001 vs. cytokines + *S. aureus*; ^°°°^ *p* < 0.001 vs. cytokines + Tempol 0.1 mM; ^@@@^ *p* < 0.001 vs. cytokines + Tempol 0.2 mM; ^§§^ *p* < 0.01 vs. cytokines + Tempol 0.5 mM; ^^^^^ *p* < 0.001 vs. cytokines + *S. aureus* + Tempol 0.1 mM; ^δδδ^ *p* < 0.001 vs. cytokines + *S. aureus* + Tempol 0.2 mM; ^‘ ‘ ‘^ *p* < 0.001 vs. cytokines+*S. aureus* + Tempol 0.5 mM.

An MTT assay was first performed to identify effective Tempol concentrations with minimal cytotoxicity in RHE. The results showed that Tempol did not affect cell viability at any concentration, indicating the absence of cytotoxic effects compared with those of the 1% SDS group (Figure 7A). The positive control (SDS 1%) reduced cell viability to 29%, confirming the expected sensitivity and functional responsiveness of the SkinEthic RHE model to cytotoxic challenge [46].

Subsequently, the effects of Tempol were evaluated following the induction of the psoriatic‐like phenotype using a cytokine mixture. Cytokine stimulation significantly reduced cell viability compared with the untreated control (Figure 7B), suggesting a state of cellular stress associated with the induction of the inflammatory phenotype. In contrast, Tempol treatment at 0.5 mM and 1 mM significantly preserved tissue viability, whereas lower concentrations were ineffective, as shown in Figure 7B.

The protective activity of Tempol was further examined under more severe conditions mimicking psoriasis associated with *S. aureus* infection. Also, in this model, only Tempol at 0.5 mM and 1 mM also maintained RHE viability in a concentration‐dependent manner, supporting its protective efficacy even in the presence of bacterial exacerbation (Figure 7B).

In addition, bacterial adhesion was quantified as the ratio between the CFU of adhered bacteria and the initial bacterial inoculum. As reported in Figure 7C, *S. aureus* adherence was markedly increased in the psoriasis‐associated infection group compared with that in CTR tissues. Notably, treatment with Tempol at 0.5 mM and 1 mM significantly reduced bacterial adhesion, whereas lower concentrations (0.1 and 0.2 mM) failed to counteract the inflammatory and infection‐associated alterations (Figure 7C). Consistently, these lower doses were ineffective in both cytokine‐induced psoriasis conditions and in the presence of *S. aureus*, further indicating a concentration‐dependent protective effect of Tempol.

For this reason, we conducted skin barrier analyses on RHE by excluding Tempol concentrations of 0.1 and 0.2 mM.

### 3.8. Tempol Treatment Restored Epidermal Barrier‐Related Proteins in Psoriasiform RHE

To investigate whether Tempol could preserve epidermal barrier integrity in the psoriasiform RHE model, we focused on the analysis of key proteins involved in the barrier function and keratinocyte differentiation.

**Figure 8:**
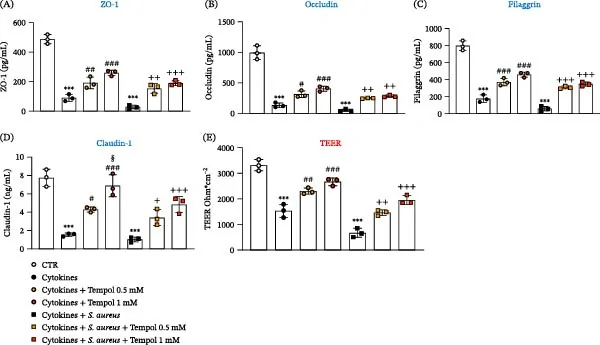
Effects of Tempol on epidermal barrier‐related proteins in psoriasiform RHE. Protein levels of ZO‐1 (A), Occludin (B), Filaggrin (C), and Claudin‐1 (D) were evaluated by ELISA in cytokine‐induced and *S. aureus*‐associated psoriasiform RHE treated with Tempol (0.5 and 1 mM). TEER was also performed (E). Data are presented as mean ± SD from three independent experiments. One‐way ANOVA followed by Bonferroni’s post hoc test.  ^∗∗∗^ *p* < 0.001 vs. CTR; ^#^ *p* < 0.05, ^##^ *p* < 0.01, ^###^ *p* < 0.001 vs. cytokines; ^+^ *p* < 0.05, ^++^ *p* < 0.01 and ^+++^ *p* < 0.001 vs. cytokines + *S. aureus*; ^§^ *p* < 0.05 vs. cytokines + Tempol 0.5 mM.

ELISA analyses demonstrated that stimulation with cytokines significantly reduced the levels of the tight junction and differentiation markers ZO‐1, Occludin, Filaggrin, and Claudin‐1 compared with CTR, indicating impairment of epidermal barrier integrity (Figure 8A–D). This reduction was even more pronounced in the cytokines + *S. aureus* group, suggesting a further worsening of barrier damage under infection‐associated conditions (Figure 8A–D).

Treatment with Tempol significantly counteracted these alterations in a concentration‐dependent manner. Indeed, both 0.5 mM and 1 mM Tempol increased the levels of all barrier‐associated proteins, with the strongest protective effect generally observed at 1 mM (Figure 8A–D). Notably, Tempol also restored ZO‐1, Occludin, Filaggrin, and Claudin‐1 levels in the *S. aureus*‐associated psoriasis model, partially reversing the additional barrier disruption caused by bacterial infection (Figure 8A–D).

Consistent with these findings, TEER measurements demonstrated a significant reduction in epithelial barrier integrity following cytokine stimulation compared with the CTR group, with a further decrease observed in the cytokines + *S. aureus* condition (Figure 8E). Treatment with Tempol at both 0.5 mM and 1 mM significantly increased TEER values under both experimental conditions, indicating improved barrier function, with the greatest effect generally observed at 1 mM (Figure 8E).

## 4. Discussion

Psoriasis is currently managed through a broad therapeutic spectrum ranging from conventional systemic agents, including methotrexate, cyclosporine, and retinoids, to targeted small molecules such as TYK2/JAK inhibitors and biologic therapies directed against TNF‐α, IL‐17, and IL‐23 pathways [47, 48]. Although these treatments have transformed disease management, important limitations remain, including incomplete responses in subsets of patients, loss of efficacy over time, safety concerns associated with chronic immunomodulation, and limited effects on oxidative damage and epidermal barrier dysfunction, both increasingly recognized as relevant pathogenic components of disease progression [49, 50].

Within this framework, pharmacological strategies capable of simultaneously targeting multiple pathogenic mechanisms may offer advantages beyond highly selective approaches.

The present study provides evidence that Tempol exerts broad protective effects across complementary psoriasiform models, accompanied by modulation of inflammatory signaling, redox imbalance, keratinocyte survival, and epidermal barrier‐associated markers. One of the principal findings of this study is that Tempol modulated the expression of key components of the TLR4/NF‐κB signaling pathway. Although psoriasis is primarily considered an IL‐23/IL‐17‐driven disease [51], increasing evidence indicates that innate immune signaling contributes significantly to disease initiation and amplification, with TLR4 emerging as a relevant upstream mediator in keratinocyte activation, cytokine release, and chronic inflammatory maintenance [52]. In the present study, Tempol modulated the expression of TLR4 pathway‐related proteins together with the restoration of IκBα expression.

Consistent with these molecular changes, Tempol significantly reduced the expression and release of TNF‐α, IL‐1β, and IL‐6 in M5‐stimulated keratinocytes, while also suppressing IL‐8, IL‐17A, and IL‐23 in the IMQ‐stimulated coculture model. Particularly noteworthy is the modulation of IL‐17A and IL‐23, cytokines central to the therapeutic mechanisms of several current biologics. Although these findings do not imply equivalence to selective cytokine‐targeting agents, they support the hypothesis that Tempol may act as a complementary pharmacological strategy capable of indirectly influencing pathways currently addressed by targeted therapies.

A second major finding concerns the modulation of redox homeostasis, characterized by increased expression of Nrf2/HO‐1 pathway components together with reduced NOX2 and NOX4 expression. Oxidative stress is increasingly recognized not merely as a consequence of inflammation but as an active pathogenic contributor linked to keratinocyte dysfunction, immune activation, and disease severity [53, 54]. Tempol enhanced Nrf2, HO‐1, and MnSOD expression while reducing NOX2 and NOX4 levels, effects accompanied by attenuation of lipid peroxidation and nitric oxide overproduction. These findings suggest that Tempol may contribute to restoring the redox balance by enhancing endogenous antioxidant defenses while reducing the expression of ROS‐generating enzymes. This interplay may contribute to the pleiotropic effects observed in the present study and may be particularly relevant in psoriasis, where inflammation and oxidative stress mutually reinforce disease progression [55].

Psoriasis is also characterized by dysregulation of the apoptotic process [56, 57]. Previous studies have reported an altered expression of apoptosis‐related proteins, including decreased Bcl‐2 and increased Bax and Bcl‐x, in psoriatic lesions, indicating an imbalance between prosurvival and proapoptotic signaling [56–58]. Consistent with its anti‐inflammatory and antioxidant properties, Tempol was associated with normalization of Bcl‐2, Bax, and p53 expression, as well as reduction of the Bax/Bcl‐2 ratio.

Although altered keratinocyte apoptosis in psoriasis remains complex and context‐dependent, these findings suggest that Tempol may contribute to preserving epithelial homeostasis under inflammatory stress conditions.

An additional translationally relevant finding is the ability of Tempol to restore epidermal barrier‐associated proteins, including ZO‐1, Occludin, Claudin‐1, and Filaggrin. These molecular findings were further supported by TEER measurements, which demonstrated improved epithelial barrier function following Tempol treatment under both cytokine‐induced and infection‐associated conditions. The concordance between barrier‐related protein expression and functional TEER assessment strengthens the evidence that Tempol contributes to preserving epidermal barrier integrity in the RHE model. In psoriasis, barrier dysfunction is increasingly recognized as an active contributor to disease exacerbation, facilitating inflammatory amplification and altered host–microbial interactions [59]. In this regard, the observed reduction in *S. aureus* adhesion under infection‐associated conditions is particularly noteworthy, suggesting that Tempol may influence not only inflammatory signaling but also epithelial resilience and host–microbiota interactions. This broader activity distinguishes Tempol from approaches exclusively focused on immune suppression.

From a pharmacological perspective, the concentration‐dependent effects observed throughout the study support a biologically coherent activity profile, with 0.5 and 1 mM consistently emerging as the most effective concentrations across inflammatory, oxidative, and barrier‐related endpoints. However, these concentrations are relatively high, and their translational feasibility remains to be established. Although topical administration represents a potential strategy for achieving higher local exposure while limiting systemic distribution, the present study did not assess temporal skin permeation, epidermal retention, local pharmacokinetics, or tissue concentrations. Therefore, it cannot presently be concluded that the effective concentrations identified in vitro are achievable in the human skin. Furthermore, the absence of cytotoxicity observed in HaCaT cells and the RHE under the present experimental conditions should not be interpreted as evidence of clinical safety. Dedicated formulation, skin permeation and retention studies, local exposure measurements, and appropriate preclinical safety assessments will be required to determine whether therapeutically relevant concentrations of Tempol can be safely achieved following topical administration.

Our findings are also consistent with growing evidence indicating that pharmacological modulation of oxidative stress‐sensitive inflammatory pathways may represent a relevant strategy in psoriasis. In support of this concept, Wogonin [60] has recently been shown to attenuate psoriasiform inflammation by reducing reactive oxygen species production and suppressing the AKT/NF‐κB signaling pathway, resulting in decreased inflammatory cytokine expression both in M5‐stimulated keratinocytes and in IMQ‐induced psoriasis‐like mice. These observations reinforce the concept that targeting redox‐dependent inflammatory signaling may provide therapeutic benefits beyond conventional immunomodulatory approaches.

The present findings should also be considered in the context of other pharmacological compounds that have shown protective effects in psoriasis‐relevant experimental models through partially overlapping molecular mechanisms. Geniposide, for example, has been reported to ameliorate psoriatic inflammation through inhibition of the TLR4/MyD88/NF‐κB p65 signaling pathway, supporting the relevance of TLR4‐dependent innate immune signaling as a pharmacological target in psoriasis [15]. Similarly, Tectorigenin has been shown to attenuate M5‐induced hyperproliferation and inflammatory responses in HaCaT keratinocytes through modulation of the TLR4/NF‐κB pathway and the NLRP3 inflammasome [61]. The latter study is particularly relevant to the present findings because it employed an M5‐stimulated HaCaT model comparable to that used in our experiments, further supporting the pharmacological relevance of targeting TLR4/NF‐κB‐associated signaling in cytokine‐stimulated keratinocytes.

A complementary point of comparison is represented by fumarate‐based pharmacological approaches. Monomethyl fumarate (MMF), an active fumarate metabolite with established relevance to psoriasis therapy, has been shown to increase total and nuclear Nrf2 levels in keratinocytes and to enhance the expression of downstream antioxidant mediators, including HO‐1 [62]. More recent experimental evidence has further demonstrated that MMF‐based treatment can modulate the interconnected Nrf2/NF‐κB network, reducing oxidative stress and inflammatory responses in both keratinocyte‐based systems and experimental psoriasis in vivo [63]. These findings are consistent with the broader concept emerging from our study that simultaneous modulation of redox homeostasis and inflammatory signaling may represent a relevant pharmacological strategy in psoriasis.

Nevertheless, important differences between these approaches should be acknowledged. The present study was not designed to establish the superiority of Tempol over Geniposide, Tectorigenin, fumarates, or other candidate antipsoriatic compounds, and direct efficacy comparisons cannot therefore be made. Rather, Tempol appears to share with these agents the ability to influence interconnected inflammatory and oxidative pathways while displaying a broader protective profile across the complementary models investigated here. In particular, in addition to modulation of TLR4/NF‐κB‐ and Nrf2‐associated signaling, Tempol improved epidermal barrier‐related protein expression and functional TEER and reduced *S. aureus* adhesion in RHE. These findings extend the comparison beyond inflammatory pathway modulation and suggest that the potential value of Tempol may also involve the preservation of epithelial barrier resilience under inflammatory and infection‐associated conditions.

Despite the promising results, some limitations should nevertheless be acknowledged. First, although multiple complementary in vitro and reconstructed epidermal models were employed, in vivo confirmation in experimental psoriasis models is required to establish therapeutic relevance under more complex physiological conditions. Second, while the data support involvement of the TLR4/NF‐κB/Nrf2 axis, validation through TLR4/NF‐κB or Nrf2‐specific inhibitor or genetic knockdown approaches was not performed and should be addressed in future studies to confirm these findings. Indeed, although Tempol increased Nrf2 expression together with downstream antioxidant mediators such as HO‐1 and MnSOD, Nrf2 nuclear translocation was not directly assessed by nuclear/cytosolic fractionation or immunofluorescence. Therefore, the present findings support modulation of Nrf2‐associated antioxidant signaling but do not definitively demonstrate the transcriptional activation of Nrf2. Future studies should assess the Nrf2 nuclear accumulation and transcriptional activity using subcellular fractionation, immunofluorescence, or other complementary approaches.

Third, NF‐κB expression was assessed at the total protein level, whereas p65 phosphorylation and nuclear translocation were not evaluated. Since phosphorylation and nuclear translocation of NF‐κB p65 represent key indicators of its activation status, the present findings should be interpreted as evidence of modulation of NF‐κB‐associated signaling components rather than as a direct demonstration of NF‐κB pathway inhibition. Future studies assessing phospho‐p65 levels and its nuclear localization will therefore be necessary to more definitively characterize the effects of Tempol on NF‐κB activation.

Another limitation of this study is that cell viability was assessed using the MTT assay, which primarily reflects cellular metabolic activity. Although MTT is a well‐established and widely used assay for estimating cell viability and cytotoxicity, future studies should corroborate these findings using complementary viability assays, such as live/dead staining.

Moreover, in vitro investigations were performed using the immortalized HaCaT keratinocyte cell line. Although this model is widely used and well established for studying psoriasis‐related mechanisms, validation in primary human keratinocytes would further strengthen the translational relevance of the findings. An additional methodological limitation concerns the use of commercial ELISA assays for the quantification of several intracellular proteins. Although the observed protein‐level changes were generally supported by concordant transcriptional findings, and key signaling proteins including NF‐κB, IκBα, and Nrf2 were additionally assessed by Western blot, not all intracellular targets evaluated by ELISA were independently validated using an orthogonal protein‐based method. Therefore, findings concerning individual intracellular proteins should be interpreted with appropriate caution, and future studies should include Western blot or other complementary protein‐level approaches to independently confirm these observations. Furthermore, keratinocyte proliferation was not directly assessed using proliferation‐specific markers such as Ki‐67 or BrdU incorporation. Since epidermal hyperproliferation represents a major hallmark of psoriasis, the absence of this endpoint limits the extent to which the present models reproduce the complete psoriatic phenotype and does not allow conclusions regarding a direct effect of Tempol on keratinocyte proliferation. Future studies incorporating proliferation‐specific analyses will therefore be required to determine whether the protective effects of Tempol also extend to the hyperproliferative component of psoriasis. In addition, the present study did not include an established antipsoriatic drug as a pharmacological positive control or a pathway‐specific inhibitor as a mechanistic comparator. Therefore, although the findings demonstrate protective effects of Tempol across the experimental models employed, they do not allow its efficacy to be directly compared with currently available antipsoriatic treatments or the specific contribution of TLR4/NF‐κB signaling to be conclusively established. Future studies should incorporate appropriate pharmacological benchmark treatments and pathway‐specific controls to address these questions. An additional methodological limitation concerns the use of GAPDH as the sole reference gene for qRT‐PCR normalization. Although GAPDH was consistently used across all experimental conditions, the inclusion and validation of multiple reference genes would provide a more robust normalization strategy. Future studies should therefore validate additional reference genes and employ multiple stable housekeeping genes for qRT‐PCR normalization.

Though the concentrations of Tempol used in this study were selected based on previous in vitro evidence and were confirmed to be noncytotoxic while preserving epithelial barrier integrity, their translational relevance following topical administration remains to be established. Pharmacokinetic studies, skin permeation analyses, and local tissue exposure assessments will be necessary to determine whether comparable concentrations can be achieved in vivo and to support the clinical translation of these findings. Finally, no a priori power analysis was performed to determine the sample size for the in vitro experiments. Although all experiments were conducted in triplicate following established practice for exploratory mechanistic studies, future in vivo and clinical studies including formal sample size estimation would further strengthen the statistical robustness of the findings.

Despite these limitations, the present findings support the concept that Tempol may act as a multitarget modulator capable of simultaneously attenuating inflammatory signaling, restoring redox homeostasis, and improving epidermal barrier integrity. Given the multifactorial pathogenesis of psoriasis, such pleiotropic activity may represent a promising adjunctive or complementary therapeutic strategy. Further translational studies will be necessary to determine whether these mechanistic effects can be leveraged for therapeutic development in psoriasis and related inflammatory skin disorders.

## 5. Conclusion and Future Perspectives

**Figure 9:**
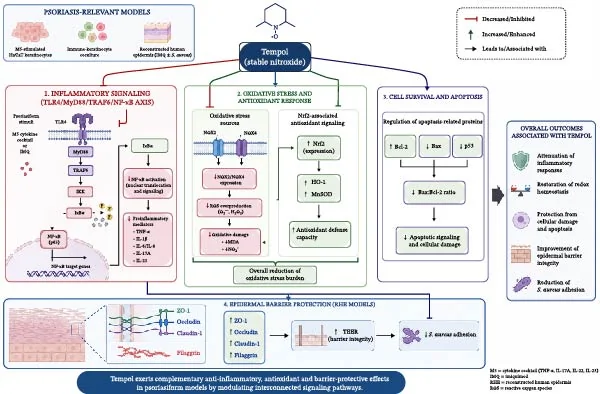
Schematic representation of the proposed pharmacological effects of Tempol in psoriasis‐relevant experimental models.

The present study provides evidence that Tempol exerts protective effects across complementary psoriasiform models through coordinated modulation of inflammatory, oxidative, and barrier‐related pathways, as recapitulated in Figure 9.

Tempol displayed a multitarget pharmacological profile that may be particularly relevant in a multifactorial disease such as psoriasis. Beyond its well‐known anti‐inflammatory activity, the ability of Tempol to reduce bacterial adhesion and improve barrier‐associated protein expression suggests potential benefits extending to epithelial resilience and host–microbiota interactions, two emerging components of psoriasis pathophysiology. In this regard, Tempol may represent a promising adjunctive candidate for the development of innovative topical approaches aimed at complementing current therapies.

Future studies should focus on validating these findings in in vivo models of psoriasis, defining the contribution of TLR4/NF‐κB/Nrf2 signaling through pharmacological or genetic approaches as well, and investigating formulation strategies capable of optimizing topical delivery, skin penetration, and local pharmacological exposure. Further studies exploring combination approaches with existing antipsoriatic therapies, as well as the potential role of Tempol in infection‐associated exacerbations or microbiota‐driven disease modulation, may also be warranted.

## Author Contributions

**Alessio Ardizzone and Sarah Adriana Scuderi**: writing – original draft. **Anna Paola Capra:** data curation, writing – review and editing. **Sarah Adriana Scuderi, Marika Lanza, and Antonio Catalfamo**: methodology. **Emanuela Esposito**: supervision. **Alessio Ardizzone and Emanuela Esposito**: conceptualization.

## Funding

This research received no external funding. Open access publishing facilitated by Universita degli Studi di Messina, as part of the Wiley‐CRUI‐CARE agreement.

## Disclosure

All authors have read and agreed to the published version of the manuscript. Moreover, all authors agree to be accountable for the content and conclusions of the article.

## Ethics Statement

The authors have nothing to report.

## Consent

The authors have nothing to report.

## Conflicts of Interest

The authors declare no conflicts of interest.

## Supporting Information

Additional supporting information can be found online in the Supporting Information section.

## Supporting information


**Supporting Information** Table S1: List of primers used for RT‐qPCR.

## Data Availability

All data of this study are included in this article.
