# Supplementary material for: Tempol Ameliorates Psoriatic Skin Inflammation: Evidence of TLR4/NF‐κB/Nrf2 Axis Modulation
Source: Mediators Inflamm. 2026 Sep 12;2026:5063546. doi: 10.1155/mi/5063546 (PMC13570453; doi:10.1155/mi/5063546)
Supplement: Supplementary file 1 [file MI-2026-5063546-s001.docx]

| Gene | Forward | Reverse |
| --- | --- | --- |
| TNF-α | CTCTTCTGCCTGCTGCACTTTG | ATGGGCTACAGGCTTGTCACTC |
| IL-1β | CCACAGACCTTCCAGGAGAATG | GTGCAGTTCAGTGATCGTACAGG |
| IL-6 | AGACAGCCACTCACCTCTTCAG | TTCTGCCAGTGCCTCTTTGCTG |
| NF-κB | TATTTCAACCACAGATGGCACT | AGCAAAGGCAATACATACACTT |
| IκBα | TCCACTCCATCCTGAAGGCTAC | CAAGGACACCAAAAGCTCCACG |
| TLR4 | CCCTGAGGCATTTAGGCAGCTA | AGGTAGAGAGGTGGCTTAGGCT |
| Myd88 | GAGGCTGAGAAGCCTTTACAGG | GCAGATGAAGGCATCGAAACGC |
| TRAF6 | CAATGCCAGCGTCCCTTCCAAA | CCAAAGGACAGTTCTGGTCATGG |
| Nrf2 | CACATCCAGTCAGAAACCAGTGG | GGAATGTCTGCGCCAAAAGCTG |
| MnSOD | CTGGACAAACCTCAGCCCTAAC | AACCTGAGCCTTGGACACCAAC |
| HO-1 | CCAGGCAGAGAATGCTGAGTTC | AAGACTGGGCTCTCCTTGTTGC |
| NOX2 | AGCTATGAGGTGGTGATGTTAGTGG | TGCAATATTTGTACCAGACTGACTTGAG |
| NOX4 | CTCAGCGGAATCAATCAGCTGTG | AGAGGAACACGACAATCAGCCTTAG |
| Bcl-2 | GAGGATTGTGGCCTTCTTTGAG | AGCCTCCGTTATCCTGGATC |
| BAX | GGACGAACTGGACAGTAACATG | GCAAAGTAGAAAAGGGCGACA |
| P53 | AGAGTCTATAGGCCCACCCC | GCTCGACGCTAGGATCTGAC |
| GAPDH | GTCTCCTCTGACTTCAACAGCG | ACCACCCTGTTGCTGTAGCCAA |

**Supplementary Table 1. List of primers used for RT-qPCR**
